# In situ Simulation of Thermal Reality

**DOI:** 10.34133/research.0222

**Published:** 2023-09-22

**Authors:** Peng Jin, Jinrong Liu, Fubao Yang, Fabio Marchesoni, Jian-Hua Jiang, Jiping Huang

**Affiliations:** ^1^ Department of Physics, State Key Laboratory of Surface Physics, and Key Laboratory of Micro and Nano Photonic Structures (MOE), Fudan University, Shanghai 200438, China.; ^2^ Center for Phononics and Thermal Energy Science, Shanghai Key Laboratory of Special Artificial Microstructure Materials and Technology, School of Physics Science and Engineering, Tongji University, Shanghai 200092, China.; ^3^ Department of Physics, University of Camerino, Camerino 62032, Italy.; ^4^ Suzhou Institute for Advanced Research, University of Science and Technology of China, Suzhou 215123, China.

## Abstract

Simulated reality encompasses virtual, augmented, and mixed realities—each characterized by different degrees of truthfulness in the visual perception: “all false,” “coexistence of true and false,” and “difficult distinction between true and false,” respectively. In all these technologies, however, the temperature rendering of virtual objects is still an unsolved problem. Undoubtedly, the lack of thermal tactile functions substantially reduces the quality of the user’s real-experience perception. To address this challenge, we propose theoretically and realize experimentally a technological platform for the in situ simulation of thermal reality. To this purpose, we design a thermal metadevice consisting of a reconfigurable array of radiating units, capable of generating the thermal image of any virtual object, and thus rendering it in situ together with its thermal signature. This is a substantial technological advance, which opens up new possibilities for simulated reality and its applications to human activities.

## Introduction

Simulated reality [1–5], also known as immersive computing technology, provides a computer-generated digital landscape that allows users to enter and operate in a simulated environment. It proved to be a powerful tool in a variety of fields, including studio entertainment [6–8], art design [9], education [10], business [11], medical science [12], aerospace [13], and other industrial sectors [14,15]. Over the past few decades, core technologies such as video coding optimization [16], controlled rehabilitation scenarios [17], and more emerging technologies in, e.g., car driving and online meeting [18] have been increasingly applied to visually simulated reality. However, to achieve truly immersive simulated reality, visual experiences alone are not sufficient. The system must integrate various forms of sensory feedback, including crucially thermal sensing, to enhance the realism of the virtual perception. Recent advancements have underscored the potential of thermal technology in augmenting reality [19]. Nonetheless, a chasm still exists between state-of-the-art thermal technology, which holds promise for developing a deeper level of immersive entertainment, and the thermal devices currently used in augmented reality applications.

Ideally, simulated reality technology should recreate all perceptive functions of the users. Indeed, the expected success of simulated reality lies in its ability to offer comprehensive and real-time experiences, which necessarily include, besides interactive images and sounds [20–22], also the sense of touch [23–26] and other sensations. One of the most critical tactile functions of the human sensory system is thermoception, or the ability to sense temperature, which can significantly enhance a user’s interaction with the external environment. Therefore, many attempts have been made to recreate thermal sensing. Incipiently, researchers found that thermoception is impacted by visual perceptions [27]. For example, the association of colors to different temperatures [28], for instance, red with warmth and blue with cold, can be leveraged to enhance the realism of simulated thermal experiences. Also, by connecting infrared cameras with visual displays [29,30], a thermal infrared vision is rendered for the user’s view, manipulating thermoception in virtual reality. Recently, thermal haptic technology has found its way into various industrial applications. Devices like “Thermovr” resort to Peltier devices to simulate the heating/cooling sensation [31]. “Ambiotherm” and “Season Traveller” products provide wearable thermal haptics to enhance ambient temperature [32,33], giving a realistic convective sensation. However, despite these advancements [27–35], accurately reproducing thermal perception in simulated reality remains a challenge. In particular, virtual and actual objects in simulated reality must retain their own temperature and emit thermal radiation according to the Stefan–Boltzmann law. The advances thus far have primarily addressed ex situ settings, which leaves the in situ simulation of thermal reality a still open challenge.

**Fig. 1.**
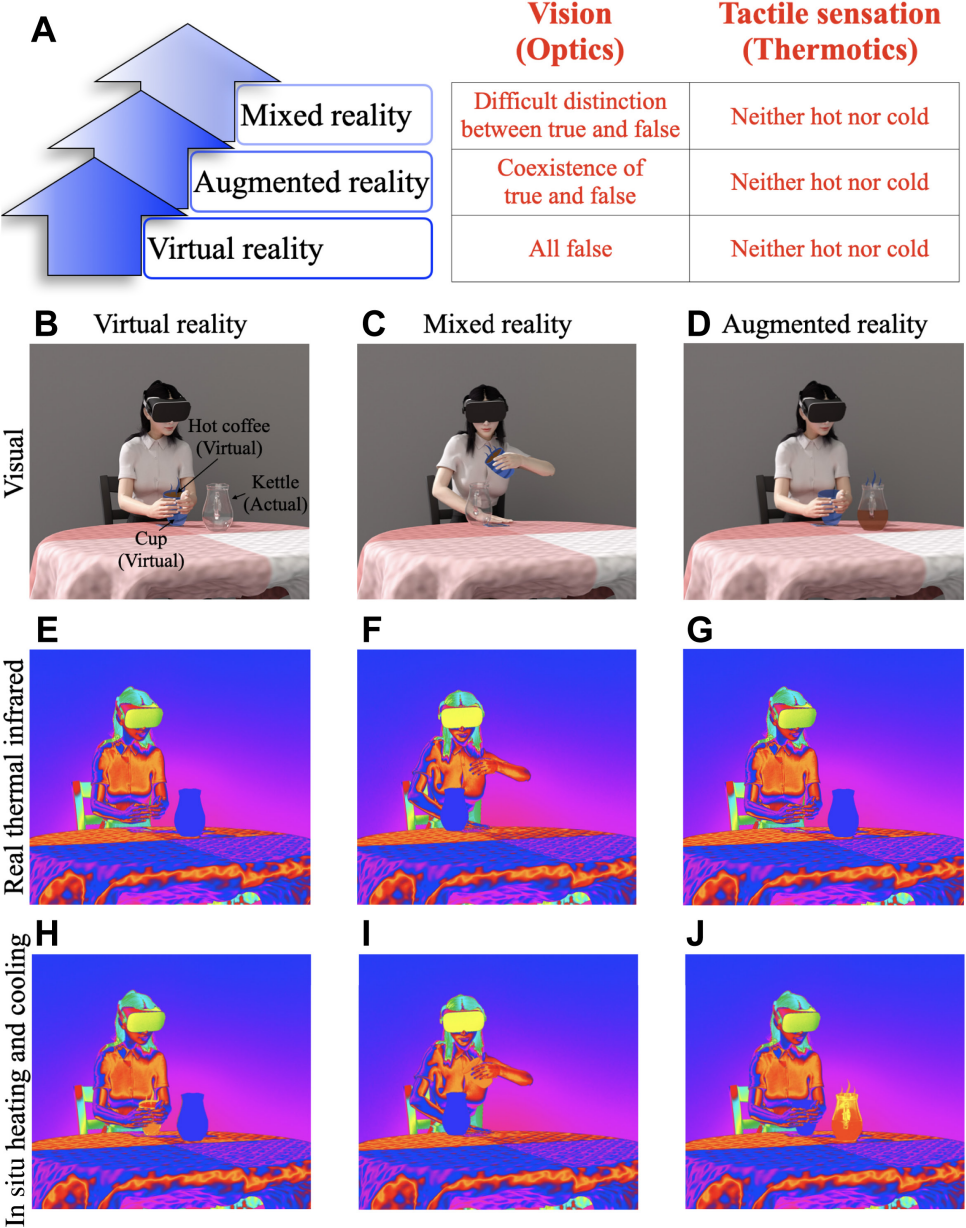
Schematic graph for heating/cooling simulated realities in situ. (A) Characteristics of visual (optics) and tactile (thermotics) experience in simulated realities. (B to D) Three visual scene renderings in simulated realities. (E to G) The real thermal infrared scenes corresponding to (B) to (D). (H to J) Same as (E) to (G), but with added in situ thermal rendering. Note that the cup and the hot coffee in (B) to (D) are virtual objects.

To address this challenge, here we develop an advanced technological platform that allows in situ thermal rendering of virtual objects within simulated reality environments. The proposed setting recreates thermal sensing and can be incorporated in thermal infrared vision. To illustrate this concept, we first present thermal infrared renderings of virtual, augmented, and mixed reality scenes through an example (Fig. 1). To achieve our goal, we design a metadevice capable of generating infrared patterns of arbitrary objects. As a proof of concept, we demonstrate experimentally that a variety of patterns can be generated in our reconfigurable thermal metadevice. Switching between different patterns was obtained by electrically reconfiguring the thermal metadevice in a programmable and computer-controlled manner. This work opens up a path toward unprecedented immersive experience with in situ thermal tactile sensation.

## Results

### Concept

Figure 1A summarizes the main characteristics of visual sensing in simulated realities. In virtual reality, computer-generated virtual objects are easily distinguishable from actual objects due to current imaging technology limitations. Augmented reality allows for more interaction between virtual objects and the natural environment, with virtual objects following the physical laws of the natural world. Mixed reality takes this a step further, offering a more natural interaction between humans and virtual objects, with virtual objects responding to human actions in a real environment. However, as mentioned above, a key element still missing in the current simulated reality settings is the thermal perception of virtual objects and environments. The proposed technology of “in situ simulation of thermal realities” aims at overcoming such a limitation. We have selected specific scenarios in simulated realities to showcase the expected scenarios obtained using our technology. Figure 1B to D illustrates a scene where a user is sitting in front of a table (actual) and creates a cup of hot coffee (virtual) in her hand using a simulated reality device (Fig. 1B). She then pours the hot coffee into a kettle (actual) on the table (Fig. 1C), and the hot coffee fills the kettle (Fig. 1D). It is worth emphasizing that the creation of virtual objects in Fig. 1B, the interaction between the user and virtual objects in Fig. 1C, and the interaction between virtual objects and the natural environment in Fig. 1D exhibit the characteristics of virtual, mixed, and augmented reality, respectively. Figure 1E to G illustrates the real thermal infrared scenes of Fig. 1B to D, where virtual objects (cup and coffee) do not emit thermal radiation. Vice versa, based on the technology of “in situ simulation of thermal realities,” virtual objects (cup and coffee) do generate thermal radiation corresponding to their temperature, as shown in Fig. 1H to J.

### Experimental Realization

The proposed approach to the in situ simulation of thermal realities consists of 3 steps. In the first step, a radiating metadevice is developed, based on the “thermotics” technology [36–48], capable of generating the thermal infrared pattern of an arbitrary object [49–60]. In the second step, using the existing computer rendering technology, the generated thermal signals are seamlessly associated with the virtual objects in the simulated reality scene. In the third step, the corresponding thermal tactile sensing of virtual objects is recreated in situ by means of an appropriate hardware. The present work focuses on the experimental implementation of the first step.

**Fig. 2.**
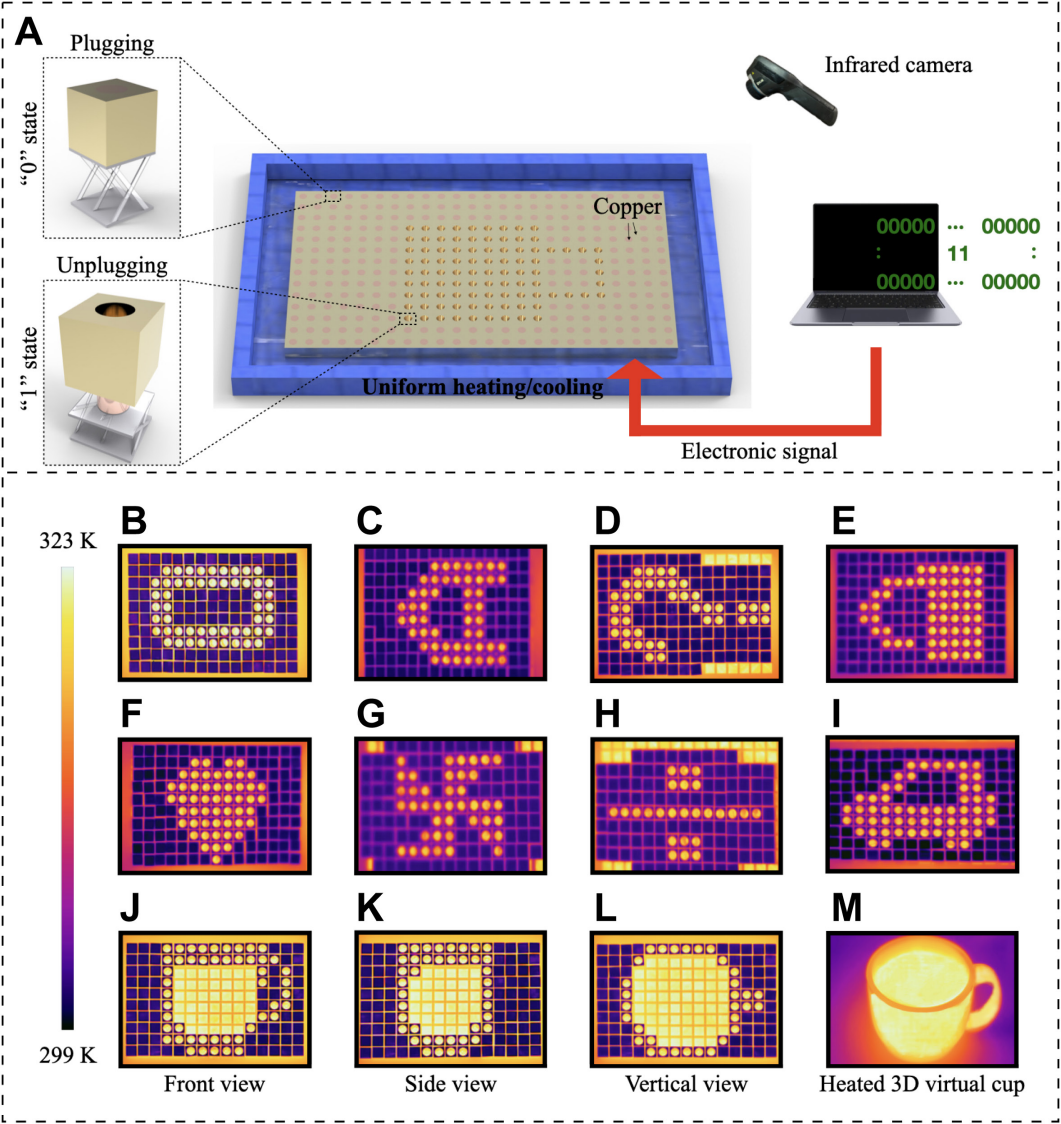
Experimental setup and measurements. (A) Schematic experimental setup. (B to I) Measured 2D thermal infrared patterns of the characters “0,” “A,” “?,” a lock, a heart, a windmill, the division sign, and a car. (J to L) Measured 2D thermal infrared views of a cup. (M) 3D virtual rendering of a heated cup obtained combining (J) to (L).

The experimental setup, sketched in Fig. 2A, consists of a metadevice in the form of a square array of programmable cubic units, placed on a uniform heat source. A computer controls electronically the plugging (“0” state) and unplugging (“1” state) of the cylinder (copper) at the center of each unit (copper), thus tuning the effective emissivity of the unit. More refined thermal patterns can be obtained by partial unplugging of the unit cylinders. We start by artificially configuring the initial state of the units targeted in the experiment. In principle, the effective thermal emissivity of the units can be independently and continuously adjusted. To create clearer thermal infrared patterns, we choose the “plugging” and “unplugging” units with the minimum and maximum emissivity, respectively. More details about the underlying theoretical mechanisms and the experimental procedures are reported in Figs. S1 and S2. As a test, we then experimentally generated the 2-dimensional (2D) thermal infrared patterns plotted in Fig. 2B to I.

### Scheme for Virtual Object Rendering

For a 3D scenario, we selected a cup as a model and generated the front, side, and vertical views of its infrared patterns shown in Fig. 2J to L. We manually merged the three 2D thermal infrared patterns (or temperature profiles) of a heated cup into the 3D view of Fig. 2M, namely, an in situ thermal image of the cup. In principle, one can develop an image processing algorithm to automatically perform such a procedure. Such a technique could streamline the process and increase efficiency in rendering virtual objects.

To begin with, the generated thermal infrared signals must be transmitted to a virtual reality setup. Our metadevice is the device used to generate the real thermal infrared signals of virtual objects. The generated signals are recorded by a thermal infrared camera. The effective temperature distributions of the metadevice infrared patterns (or say, thermal images) are then transmitted to a microcomputer system via a wire or a local-area network for further processing, namely, the 3D rendering of the virtual object. It is worth noting that the transmission of thermal signals from the infrared camera to a microcomputer, like a Raspberry Pi, has been successfully tested in a recent work [61]. In Fig. 2M, the temperature of the “hot liquid” pattern happens to be close to that of the heating plate, due to the high thermal conductivity of copper (400 W m ^−1^ K ^−1^), used as substrate for the metadevice. Of course, the temperature of the metadevice can be varied at will.

**Fig. 3.**
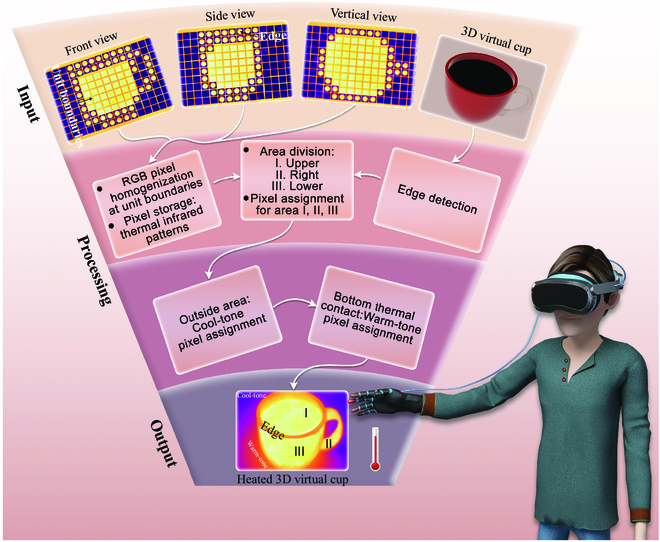
Image processing. Flowchart of an image processing algorithm for the thermal rendering of a virtual object.

An example of image processing algorithm is illustrated in Fig. 3. It consists of 3 main steps: image input, processing, and output. We have three 2D thermal infrared patterns and one 3D view of a cup model as input images. As shown in the upper part of Fig. 3, there are gaps between the units of the metadevice so that a thermal signal noise will be added at the unit boundaries for smoothing. Indeed, a task of image processing is to homogenize the RGB pixel along the unit boundaries by conveniently averaging the neighboring pixels and store the digital thermal views of the cup thus obtained. To any 3D model, there corresponds a 2D projection rendering taken along an arbitrary direction. We only need to cycle the direction as a variable to obtain the omnidirectional projection rendering. Here, we restricted ourselves to the projection of a 3D cup in one particular direction (see Fig. 3). To generate the virtual output image, we performed edge detection of the 3D cup image and recorded three 2D views of the cup thermal profile. The 3D model was divided into closed areas: the upper (I), right (II), and lower (III) ones. Next, each such area was assigned the corresponding RGB pixels extracted from the thermal infrared patterns. To the area outside the cup, we assigned a set of cool-tone RGB pixels. As in general the heated 3D virtual cup is in thermal contact with its environment at the bottom, we assigned a set of warm-tone RGB pixels to the area around the cup’s bottom. The resulting image is the heated 3D virtual cup in the lower part of Fig. 3.

### Scheme to Generate a Tactile Sensing of Heating/Cooling Virtual Objects

To enable tactile thermal sensing of the generated virtual objects, one option presently under testing is to attach thermoelectric heat/cold sources [62,63] to a wearable virtual reality glove. The thermal infrared signals from the area where the wearer’s fingers touch the virtual object represent real temperatures. The virtual reality hardware can then quickly calculate the corresponding working power of the glove’s thermoelectric sources in the contact area and thus generate the corresponding temperature perception (see Fig. 3). This in situ procedure requires a minimal power consumption. To read the thermal infrared signals from the touched virtual object, we have 2 strategies. The first involves equipping the virtual reality glove with microsensors that can capture the thermal infrared signals of the contact area with the virtual object. The second strategy is to record the glove’s coordinates in the 3D coordinate system of the virtual scene generated by the glove itself. The glove only reads and elaborates the thermal infrared signals associated with the spatial coordinates of its surface.

The in situ simulation of thermal reality is a noncontact human–computer interaction technology that provides a novel way of interaction by simulating real thermal perception. In our system, the user’s hand can hover over virtual objects in virtual reality, and the thermal perception of the virtual objects is realized by sensing their thermal radiation. This mode of interaction not only aligns with human lifestyle habits but also provides a more realistic perception experience in augmented reality environments, thereby greatly enhancing the user’s sense of immersion.

## Discussion

Compared with eyes and ears, skin is often overlooked as a sensory interface in the context of simulated reality. As the largest organ of the human body, the skin contains many thermal receptors responsible for our temperature sensing and thus plays a key role, when one tries to extend the simulated reality experience beyond visual, auditory, and mechanical perception. Our setup for in situ simulation of thermal realities represents a substantial step forward in this direction. This will offer users a more immersive experience and pave the way for industrial innovations in this field of technology, further expanding its applications and impact.

In Supplementary Materials S1, we derive the effective thermal emissivity of the cylindrical cavities from the basic principle of energy conservation. Such principles are not limited to cylindrical cavities with the same material, meeting most application requirements. The metadevice we fabricated for the experiments presented here consisted of copper cubic units of the same geometry. That simple solution worked quite well with objects of uniform temperatures. However, one can think of regulating the hole depth of each unit continuously, and thus fine tuning their effective emissivity and radiation temperature independently of each other. Such a setup would be more suitable for generating the thermal image of objects with nonuniform temperature distributions (nonuniform heating/cooling). The outcome of a finite-element simulation for one such case is reported in Fig. S3. Moreover, uniform heating/cooling the bottom of the metadevice causes all its units to be heated/cooled simultaneously, which makes their temperature control quite inconvenient. In the next future, we consider achieving full temperature control of the metadevice units by resorting to voltage-driven thermoelectric heat/cold sources [62]. As a result, nonuniform thermal images of virtual objects would span over a wider, more adjustable radiation-temperature range.

## Materials and Methods

See concrete experimental details in Supplementary Materials S2.

## Data Availability

All data needed to evaluate the conclusions in the paper are present in the paper and/or the Supplementary Materials.

## Supplementary Materials

Supplementary 1 Supplementary Materials S1 to S3 Figs. S1 to S3 Supplementary References
