# Supplementary material for: In situ Simulation of Thermal Reality
Source: Research (Wash D C). 2023 Sep 22;6:0222. doi: 10.34133/research.0222 (PMC10516180; doi:10.34133/research.0222)
Supplement: Supplementary 1 — Supplementary Materials S1 to S3 Figs. S1 to S3 Supplementary References [file research.0222.f1.pdf]

Supplementary Materials for "*In-situ* simulation of thermal reality"

Peng Jin,[1,*] Jinrong Liu,[1,*] Fubao Yang,[1] Fabio Marchesoni,[2,3] Jian-Hua Jiang,[4] and Jiping Huang[1,†]

[1]*Department of Physics, State Key Laboratory of Surface Physics, and Key Laboratory of Micro and Nano Photonic Structures (MOE), Fudan University, Shanghai 200438, China*

[2]*Center for Phononics and Thermal Energy Science, Shanghai Key Laboratory of Special Artificial Microstructure Materials and Technology, School of Physics Science and Engineering, Tongji University, Shanghai 200092, China*

[3]*Department of Physics, University of Camerino, Camerino 62032, Italy*

[4]*Suzhou Institute for Advanced Research, University of Science and Technology of China, Suzhou 215123, China*

[*] These authors contributed equally to this work.

[†] jphuang@fudan.edu.cn

S1. Effective emissivity of cylindrical cavities

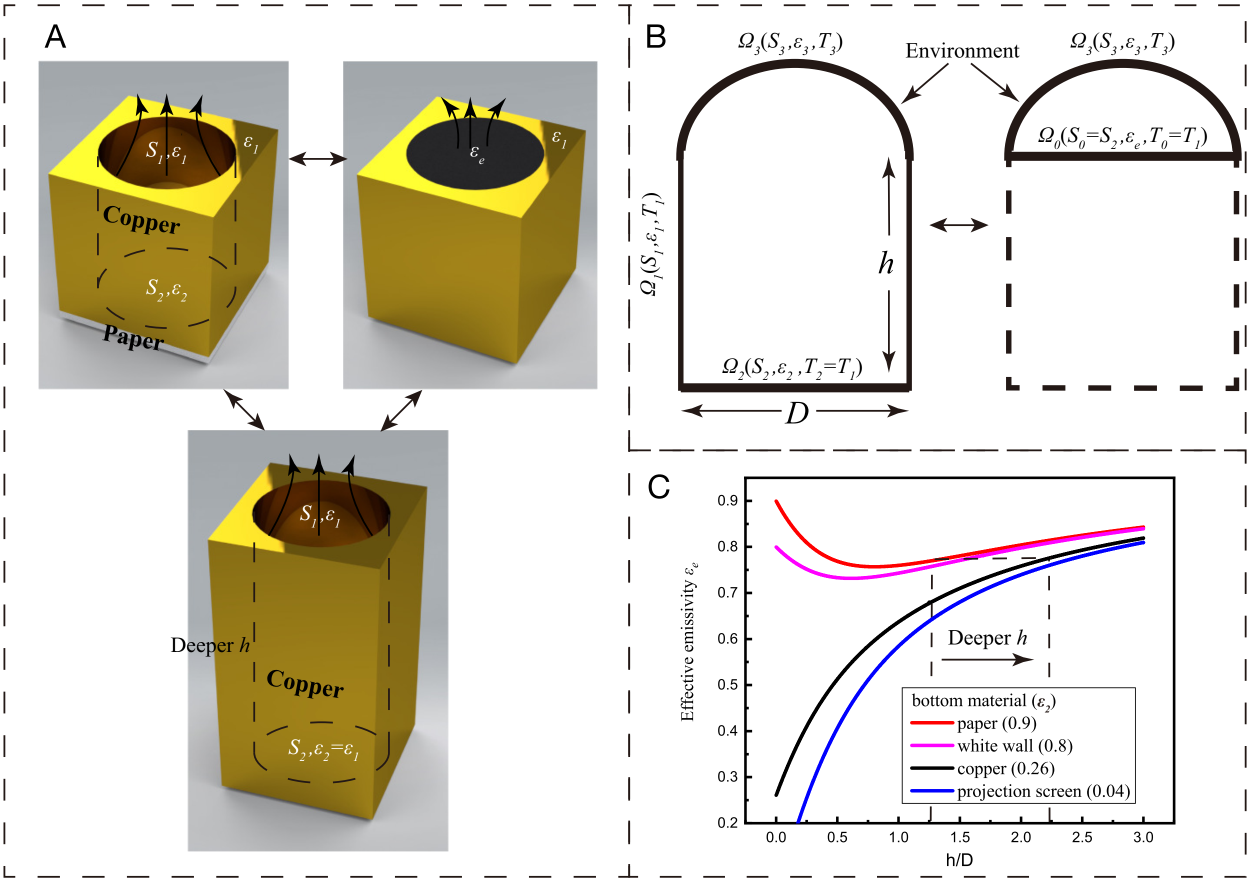

FIG. S1. The effective emissivity of cylindrical cavities. (A) Schematic of different cylindrical cavities. The effective emissivity, ε_e, of a cylindrical hole drilled through a copper slab placed on a paper sheet is equivalent to that of a surface of the same cross-section and emissivity ε_e, or to that of a cylindrical hole of appropriate depth drilled in solid copper. (B) Side view of the cylindrical cavity used in the calculation of the effective emissivity of Eq. (S5). The effective emissivity of the cavity with surfaces Ω_1 and Ω_2, respectively of emissivity ε_1 and ε_2, coincides with that of a surface Ω_0 of emissivity ε_e. (C) Comparison of the effective emissivity, ε_e, of projection-screen-bottom ($\varepsilon_2 = 0.04$, blue curve), copper-bottom ($\varepsilon_2 = 0.26$, black curve), white-wall-bottom ($\varepsilon_2 = 0.8$, purplish red curve), and paper-bottom ($\varepsilon_2 = 0.9$, red curve) cylindrical cavities.

When the bottom and wall of a cylindrical cavity of diameter D are made of the same material, one can easily calculate the cavity effective emissivity [1],

$$\varepsilon_e = \left[1 + \frac{S_2}{S_1 + S_2}\left(\frac{1}{\varepsilon} - 1\right)\right]^{-1}, \tag{S1}$$

where $S_1 = \pi D h$ and $S_2 = \pi D^2/4$ are the areas of the lateral and bottom surfaces, and ε is the material emissivity. One can tune the effective emissivity of the cavity by varying its depth. However, in many applications the bottom and the wall of the cavity can be made of different materials or kept at different temperatures, so that the radiation from the bottom and the wall are also different (Fig. S1A). Therefore, we need to generalize Eq. (S1) to the case of cavities delimited by N surfaces with different radiation properties [2]. For the k-th surface, Ω_k (with k=1,2,…,N), of area S_k and temperature T_k, we have

$$\sum_{j=1}^{N}\left(\frac{\delta_{kj}}{\varepsilon_j} - F_{k-j}\frac{1-\varepsilon_j}{\varepsilon_j}\right)\frac{Q_j}{S_j} = \sum_{j=1}^{N}\left(\delta_{kj} - F_{k-j}\right)\sigma T_j^4, \tag{S2}$$

where δ_{kj} is the Kronecker symbol, $F_{k-j} = (1/S_k)\int_{S_k}\int_{S_j}\cos\varphi_k\cos\varphi_j/(\pi r^2)\,\mathrm{d}S_k\,\mathrm{d}S_j$ are configuration factors that represent the energy radiated per unit of area by the surface Ω_k and absorbed by the surface Ω_j. Here, φ_k (φ_j) is the angle the normal to $\mathrm{d}S_k$ ($\mathrm{d}S_j$) form with the line connecting the two surface elements and r is their distance. Q_j is the incident radiation power on Ω_j, T_j the temperature of Ω_j, and σ denotes the Stefan–Boltzmann constant. For the geometry of Fig. S1B, the relevant Eq. (S2) reads

$$\begin{aligned}
\left(\frac{1}{\varepsilon_1} - F_{1-1}\frac{1-\varepsilon_1}{\varepsilon_1}\right)\frac{Q_1}{S_1} - F_{1-2}\frac{1-\varepsilon_2}{\varepsilon_2}\frac{Q_2}{S_2} - F_{1-3}\frac{1-\varepsilon_3}{\varepsilon_3}\frac{Q_3}{S_3} &= \sigma\left[(1-F_{1-1})T_1^4 - F_{1-2}T_2^4 - F_{1-3}T_3^4\right],\\
-F_{2-1}\frac{1-\varepsilon_1}{\varepsilon_1}\frac{Q_1}{S_1} + \frac{1}{\varepsilon_2}\frac{Q_2}{S_2} - F_{2-3}\frac{1-\varepsilon_3}{\varepsilon_3}\frac{Q_3}{S_3} &= \sigma(-F_{2-1}T_1^4 + T_2^4 - F_{2-3}T_3^4),\\
-F_{3-1}\frac{1-\varepsilon_1}{\varepsilon_1}\frac{Q_1}{S_1} - F_{3-2}\frac{1-\varepsilon_2}{\varepsilon_2}\frac{Q_2}{S_2} + \left(\frac{1}{\varepsilon_3} - F_{3-3}\frac{1-\varepsilon_3}{\varepsilon_3}\right)\frac{Q_3}{S_3} &= \sigma\left[-F_{3-1}T_1^4 - F_{3-2}T_2^4 + (1-F_{3-3})T_3^4\right].
\end{aligned} \tag{S3}$$

In Fig. S1B, the radiating surfaces Ω_1 and Ω_2 can be replaced by one equivalent circular surface, Ω_0, with the same diameter and temperature of the bottom surface. The surface Ω_3 with an area of S_3, equivalent to the external environment, is a cap covering cavity opening for enclosure analysis, so that we can calculate the transferring energy from the hole/surface to the environment. The corresponding system of Eqs. (S3) is then replaced by

$$\begin{aligned}
\frac{1}{\varepsilon_2}\frac{Q_0}{S_2} - F_{0-3}\frac{1-\varepsilon_3}{\varepsilon_3}\frac{Q_3}{S_3} &= \sigma(T_1^4 - F_{0-3}T_3^4),\\
-F_{3-0}\frac{1-\varepsilon_e}{\varepsilon_e}\frac{Q_0}{S_2} + \left(\frac{1}{\varepsilon_3} - F_{3-3}\frac{1-\varepsilon_3}{\varepsilon_3}\right)\frac{Q_3}{S_3} &= \sigma\left[-F_{3-0}T_1^4 + (1-F_{3-3})T_3^4\right].
\end{aligned} \tag{S4}$$

By making use of the reciprocity of the configuration factors, F_{i-j}, we eliminate Ω_3 from Eqs. (S4) and thus determine the cavity effective emissivity

$$\varepsilon_e = -\frac{A_0 + A_1\frac{S_1}{S_2}}{B_0 + B_1\frac{S_1}{S_2} + B_2(\frac{S_1}{S_2})^2}, \tag{S5}$$

where $A_0 = [\varepsilon_1(2F_{1-0} - 1) - 2F_{1-0}][\varepsilon_2(F_{2-0} - 1) - F_{2-0}]$, $A_1 = (\varepsilon_2 - 1)F_{1-0}[\varepsilon_1(F_{1-0} - 1) - F_{1-0}]$, $B_0 = \varepsilon_2 F_{2-0}[\varepsilon_1(2F_{1-0} - 1) - 2F_{1-0}]$, $B_1 = F_{1-0}[\varepsilon_1\varepsilon_2(F_{1-0} + 2F_{2-0} - 1) - 2\varepsilon_1 F_{2-0} - \varepsilon_2 F_{1-0}]$, and $B_2 = \varepsilon_1(\varepsilon_2 - 1)F_{1-0}^2$. For $\varepsilon_1 = \varepsilon_2$, $S_1 = \pi Dh$, and $S_2 = \pi D^2/4$, Eq. (S5) boils down to Eq. (S1). Then we set $S_1 = \pi Dh$, $S_2 = \pi D^2/4$, and $F_{2-0} = [x^2 - \sqrt{x^2(x^2+4)} + 2]/2$ with $S_1 F_{1-0} = S_2 F_{2-1}$ [2]. The corresponding curves ε_e versus the cavity aspect ratio, h/D, are plotted in Fig. S1C for four cases: (i) copper wall and projection-screen bottom with $\varepsilon_1 = 0.26$ and $\varepsilon_2 = 0.04$; (ii) copper wall and copper bottom with $\varepsilon_1 = \varepsilon_2 = 0.26$; (iii) copper wall and white-wall bottom with $\varepsilon_1 = 0.26$ and $\varepsilon_2 = 0.80$; (iv) copper wall and paper bottom with $\varepsilon_1 = 0.26$ and $\varepsilon_2 = 0.90$, respectively. Notice that in the case of higher emissivity bottom, (iv), the effective emissivity of the cavity increases sharply with lowering h/D.

S2. Design and characterization of the radiating metadevice

Our experimental setup is shown in Fig. S2A. The designed metadevice is a 10×15 array of copper cubes $1 \times 1 \times 1$ cm^3 in size. The effective emissivity of the solid cubic units was measured to be 0.26. To realize higher emissivity units, we drilled a hole 0.8 cm in diameter across some of the copper cubes. Units of both types were arranged on a clear acrylic lattice and then placed on a sheet of matte paper with effective emissivity of 0.90. The metadevice thus obtained is finally placed on a heating plate set at 50 °C. The temperatures of the paper sheet and copper units can be assumed to be the same as the heating plate. According to Eq. (S5), the effective emissivity of the hollow copper cube on paper is 0.77, which would be equivalent to that of a cavity with copper bottom of same cross-section but $h = 1.8$ cm deep, as illustrated Fig. S1C. A thermal infrared camera Fotric 430 was placed 0.6 m above the metadevice to record the spatial distribution of the surface temperature. The temperature pattern reported in Fig. S2B was obtained from the camera readings for a homogeneous surface emissivity, $\varepsilon = 0.98$. By rescaling the temperature of each array unit for its calculated effective emissivity, ε_e, the array temperature turned out to be uniform and close to the temperature, 50°C, of the heating plate (see Fig. S2C). This result validates our calculations for the effective emissivity in Eqs. (S2)-(S5). Finally, by arranging solid copper cubes with $\varepsilon_e = 0.26$ and paper-bottom hollow copper cubes with $\varepsilon_e = 0.77$, our metadevice can be used to generate the 2D thermal infrared patterns of any targeted virtual object, as illustrated in Fig. S2B.

S3. Finite-element simulation of a thermal infrared pattern with non-uniform temperature

The metadevice used in our experiments was made of two kinds of cubic units, solid and hol-

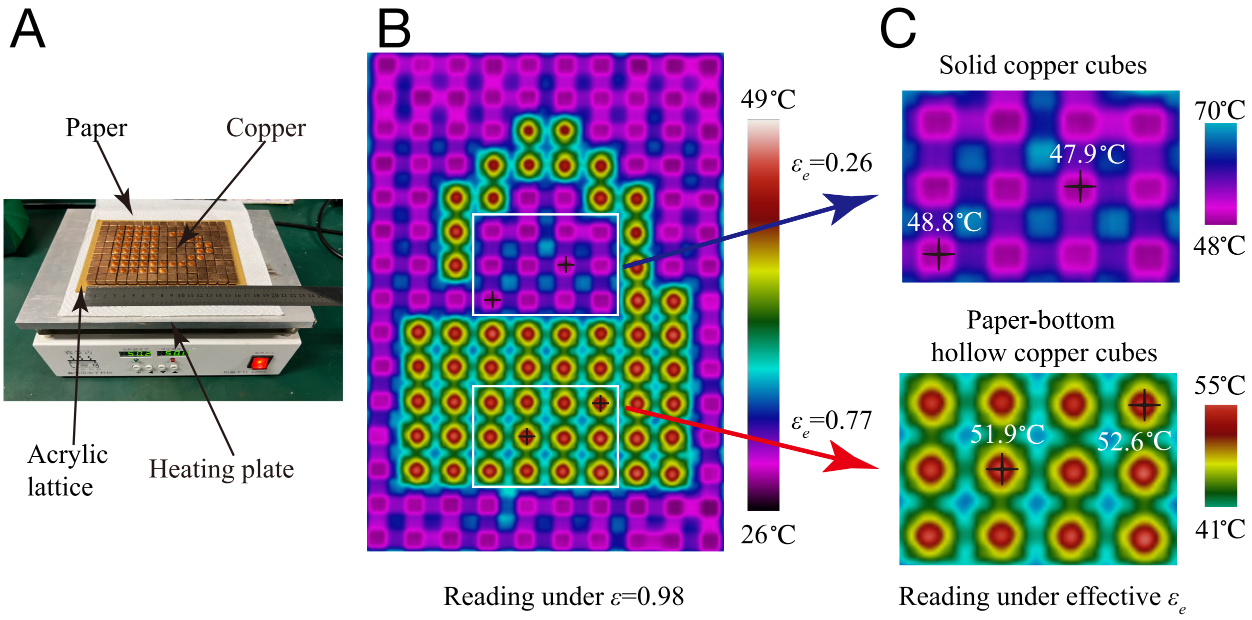

FIG. S2. Experimental details and validation. (A) Photograph of the experimental setup. (B) Thermal infrared pattern of a padlock. Camera temperature reading for a preset homogeneous surface emissivity, $\varepsilon = 0.98$. (C) The corresponding temperature readings rescaled for the effective emissivity of the two types of units, $\varepsilon_e = 0.26$ (copper-bottom) and 0.77 (paper-bottom). All rescaled temperatures come close to the temperature of the heating plate (set at 50°C.)

low. In preparation for future work, we also performed finite-element simulations of the thermal infrared patterns, we expect to generate by using units of tunable depth (stacked by a certain number of units with unit thickness in each hollow). An example is illustrated in Fig. S3. The simulation box was a 15×10 2D array of copper cubes $1 \times 1 \times 1$ cm^3 in size, like in the experiment of S2. We considered the case of just two types of metadevice units: solid copper cubes (thermal conductivity $\kappa = 400$ W m^{-1} K^{-1}), and hollow copper cubes with a cylindrical cavity of fixed diameter, $D = 0.8$ cm but tunable depth, h. Then we artificially arranged these units into the pattern of a cup, like in Fig. S3A. The bottom surface of the box was modeled as a homogeneous heat source at fixed temperature of 323.15 K (50 °C). The upper surface of the box and the walls and bottoms of the cube cavities were treated as open boundaries with emissivity $\varepsilon = 0.26$ and subject to an external air-convective heat flux with convective coefficient $h_0 = 50$ W m^{-2} K^{-1} at $T_{air} = 273.15$ K. The four sides of the box were thermally insulated. By encoding the desired environmental parameters in the finite-element algorithm (Fig. S3B), we calculated the stationary temperature profiles of the metadevice. As shown in Fig. S3C, units with deeper

cavities correspond to higher temperatures, which is consistent with the our experimental results and the theoretical discussion of S1. The final result is the thermal infrared pattern of a cup with *non-uniform* temperature.

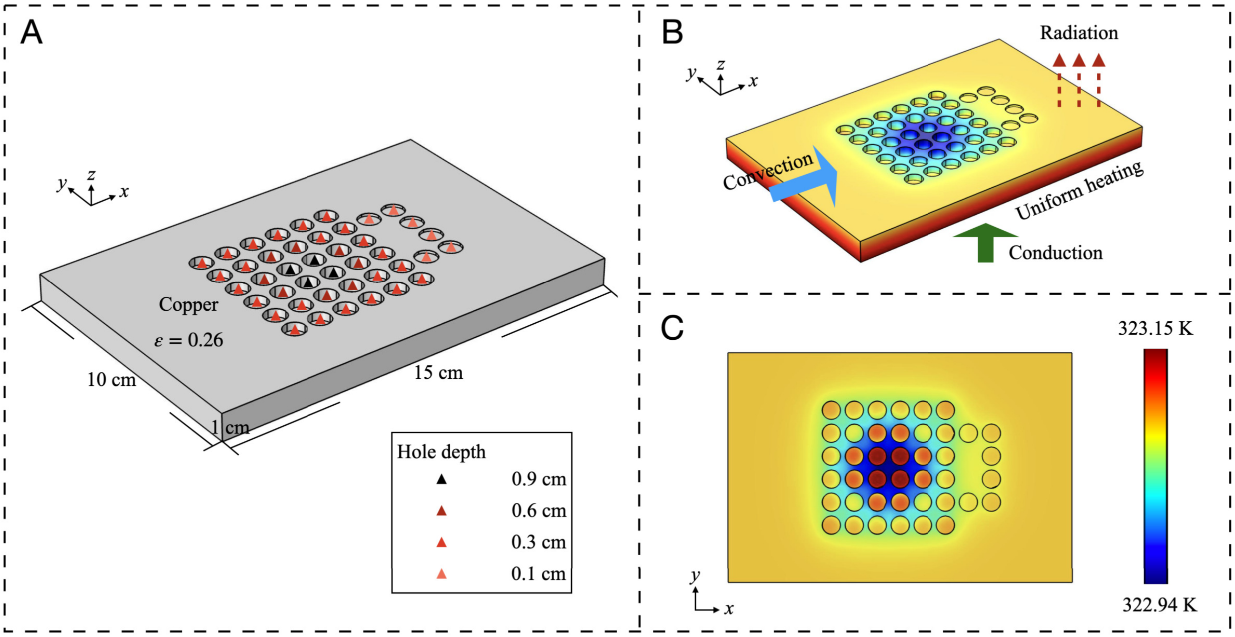

FIG. S3. Finite-element simulation scheme. (A) Size and pattern of a metadevice made of cubic units with cylindrical cavity of tunable depth. (B) Encoding of a physical environment in the finite-element algorithm. (C) Calculated thermal infrared pattern of a cup with non-uniform temperature.

[1] Wang J, Yang F, Xu L, Huang J. Omnithermal restructurable metasurfaces for both infrared-light illusion and visible-light similarity. *Phys Rev Appl.* 2020;14(1):014008.

[2] Howell J R, Menguc M P, Siegel R. *Thermal Radiation Heat Transfer.* New York (USA): CRC Press; 2015.
